# AlGaAs ^55^Fe X-ray radioisotope microbattery

**DOI:** 10.1038/srep38409

**Published:** 2016-12-06

**Authors:** S. Butera, M. D. C. Whitaker, G. Lioliou, A. M. Barnett

**Affiliations:** 1 Semiconductor Materials and Device Laboratory, School of Engineering and Informatics, University of Sussex, Brighton, BN1 9QT, UK

## Abstract

This paper describes the performance of a fabricated prototype Al_0.2_Ga_0.8_As ^55^Fe radioisotope microbattery photovoltaic cells over the temperature range −20 °C to 50 °C. Two 400 μm diameter p^+^-i-n^+^ (3 μm i-layer) Al_0.2_Ga_0.8_As mesa photodiodes were used as conversion devices in a novel X-ray microbattery prototype. The changes of the key microbattery parameters were analysed in response to temperature: the open circuit voltage, the maximum output power and the internal conversion efficiency decreased when the temperature was increased. At −20 °C, an open circuit voltage and a maximum output power of 0.2 V and 0.04 pW, respectively, were measured per photodiode. The best internal conversion efficiency achieved for the fabricated prototype was only 0.95% at −20 °C.

Radioactive sources coupled to semiconductors have received research attention for producing long life radioisotope microbatteries^1^. High-energy particles are emitted during nuclear decay of a radioisotope which is coupled to a semiconductor converter device. The semiconductor material absorbs the particles, creating electron-hole pairs and producing electrical energy over a long period of time, governed by the half-life of the chosen radioisotope. In biomedical applications, providing a power supply, that has no need of periodic recharge or replacement, is a priority for the life-quality of patients requiring implanted medical devices^2^. Microbatteries that can operate over an extended period of time are also essential in security and defense applications, for example, FPGA encryption keys with battery backed memory are used for secure exchange of information between personnel^3^. Radioisotope microbatteries are candidate devices to help meet the needs and opportunities within these research fields, as well as being potentially useful more generally in microelectromechanical system technologies (MEMS)^1^.

Nuclear (radioisotope) microbatteries provide important characteristics such as high energy density and stability; for these reasons, they can be better options than traditional power supplies (e.g. chemical batteries) in certain circumstances. Compared to narrow bandgap semiconductors, wide bandgap semiconductors are preferable as converter materials in nuclear batteries; firstly because the nuclear battery conversion efficiency increases linearly with bandgap^4^, and secondly because they can operate at elevated temperatures without cooling and are commonly radiation tolerant^5^,^6^. This makes their use possible in harsh environment conditions and reduces the likelihood of radiation damage from the integrated radioisotope or from external radiation sources. Recently, multiple types of nuclear microbattery have been reported^7^,^8^,^9^,^10^,^11^,^12^,^13^,^14^. Diamond detectors were investigated by Bormashov *et al*.^7^ for developing ^63^Ni-diamond beta-voltaic and ^238^Pu-diamond alpha-voltaic microbatteries with total battery efficiencies as high as 0.6% and 3.6%, respectively, at room temperature. GaN devices were reported by Cheng *et al*.^8^ for producing a high open circuit voltage (1.64 V) ^63^Ni-GaN beta-voltaic microbattery with a total battery efficiency of 0.98% at room temperature. Si and GaAs converter materials were used by Wang *et al*.^10^ for two different types of beta-voltaic microbattery: at 20 °C, a conversion efficiency of 0.05% was found for a ^147^Pm-Si microbattery, while 0.075% was obtained for a ^63^Ni-GaAs microbattery. SiC detectors were used by Chandrashekhar *et al*.^11^ and Eiting *et al*.^12^ at room temperature: while the first reported a ^63^Ni-SiC microbattery with at least 6% efficiency, the second demonstrated a ^33^P-SiC microbattery with 4.5% efficiency. Until recently, because of the high specific energy per Curie, mainly only alpha- and beta- emitting radioisotopes have been used in nuclear microbatteries (e.g. 0.3 μW·g·Ci^−1^·cm^−2^ for the beta emitter ^147^Pm^1^). However, recently, the electron capture X-ray emitter ^55^Fe (0.017 μW·g·Ci^−1^·cm^−2^ 1) has received research attention for nuclear microbattery use^13^,^14^. Butera *et al*. reported temperature dependent characterisation of different types of ^55^Fe radioisotope X-ray microbatteries using GaAs and Al_0.52_In_0.58_P p^+^-i-n^+^ photodiodes as conversion devices^13^,^14^: at −20 °C (the lowest temperature investigated and the temperature at which the devices had the best performance) the ^55^Fe-GaAs microbattery and the ^55^Fe-Al_0.52_In_0.58_P microbattery had internal conversion efficiencies of 9% and 22%, respectively. Although ^55^Fe has a lower energy per Curie compared to other candidate radioisotope sources, its use is attractive because of the reduced damage risk for the converter device due to the low energy photons emitted. Furthermore, the wide availability, relatively low cost, and the need for only comparatively little shielding to protect users of the microbattery from the radioisotope also contribute to making ^55^Fe an attractive option.

In this paper, an Al_0.2_Ga_0.8_As ^55^Fe radioisotope microbattery is reported for the first time. The effect of temperature on the microbattery key parameters of the X-ray-photovoltaic microbattery are described over the temperature range −20 °C to 50 °C. The choice of Al_0.2_Ga_0.8_As (bandgap = 1.67 eV at room temperature^15^) as the converter material was expected to give advantages such as lower thermally generated leakage currents than would be experience by narrower bandgap devices of same geometry. Moreover, Al_0.2_Ga_0.8_As can be grown lattice matched with GaAs, which makes its growth relatively cheap, and GaAs/AlGaAs processing techniques are more routinely available for both academia and industry when compared with alternative wide bandgap materials. These characteristics combine to make AlGaAs a desirable material for building photovoltaic cells. Recently, different types of high efficiency solar cells, based on AlGaAs structures, have been demonstrated and their properties studied^16^,^17^,^18^. The spectroscopic detection of X-ray photons using AlGaAs was firstly reported by Lauter *et al*.^19^; subsequent work by Barnett *et al*.^20^,^21^,^22^,^23^,^24^ further developed the material in a higher aluminium fraction variant (Al_0.8_Ga_0.2_As) for X-ray photon counting spectroscopy applications.

## Materials and Method

### Radioactive source and energy conversion device

The prototype X-ray photovoltaic microbattery consisted of an ^55^Fe radioisotope X-ray source (activity 230 MBq) coupled to two 400 μm diameter unpassivated p^+^-i-n^+^ Al_0.2_Ga_0.8_As mesa conversion photodiodes (D1 and D2), located on the same die. The X-ray source was positioned 5 mm away from the Al_0.2_Ga_0.8_As devices’ top surfaces. The Al_0.2_Ga_0.8_As epilayer of the devices was grown and fabricated to the Authors’ specifications by the EPSRC National Centre for III-V Technologies, Sheffield, UK. The Al_0.2_Ga_0.8_As wafer was grown on a commercial GaAs n^+^ substrate using metalorganic vapour phase epitaxy (MOVPE). The thickness of the Al_0.2_Ga_0.8_As i-layer was 3 μm; further refinement and optimisation of the growth process may enable thick, high quality Al_0.2_Ga_0.8_As epilayers to be produced in future. After growth, the wafer was processed to form mesa structures using wet etching techniques, in particular 1:1:1 H_3_PO_4_:H_2_O_2_:H_2_O solution followed by 10 s in 1:8:80 H_2_SO_4_:H_2_O_2_:H_2_O solution. Table 1 summarises the device layers, their relative thicknesses and materials. The p^+^-side Ohmic contact, consisting of 20 nm of Ti and 200 nm of Au, covered 33% of the surface of each photodiode.

The X-ray quantum efficiency (*QE*) of the Al_0.2_Ga_0.8_As ^55^Fe radioisotope X-ray photovoltaic microbattery was calculated using the Beer-Lambert law and assuming a collection efficiency of 100% in the i-layer. A top metal contact coverage (33%) of each Al_0.2_Ga_0.8_As device surface was also taken into account. *QE* values of 23% (*QE*_*Kα*_) and 18% (*QE*_*Kβ*_) were calculated for 5.9 keV and 6.49 keV photons, respectively. The Al_0.2_Ga_0.8_As X-ray linear attenuation coefficients at 5.9 keV and 6.49 keV were estimated to be 0.0788 μm^−1^ and 0.0604 μm^−1 ^25,26. At the same energies, the GaAs, Ti and Au attenuation coefficients used were from refs 25, 27.

### Experiment and measurements

**Figure 1:**
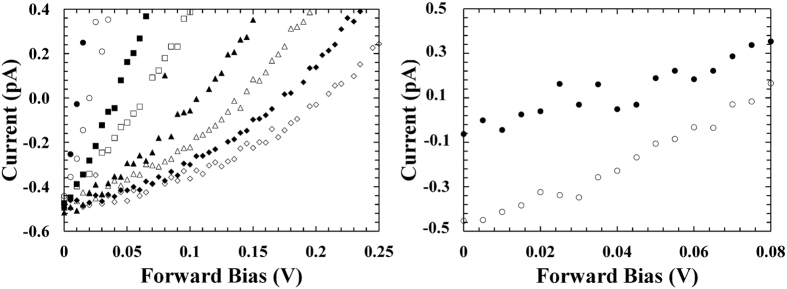
(**a**) Current as a function of applied forward bias for D1. The temperatures studied were 50 °C (filled circles), 40 °C (empty circles), 30 °C (filled squares), 20 °C (empty squares), 10 °C (filled triangles), 0 °C (empty triangles), −10 °C (filled rhombuses) and −20 °C (empty rhombuses). (**b**) Dark (filled circles) and illuminated (empty circles) current characteristics as a function of forward bias for D2 at 20 °C.

The Al_0.2_Ga_0.8_As ^55^Fe radioisotope X-ray-photovoltaic microbattery was placed inside a TAS Micro MT climatic cabinet with a dry nitrogen atmosphere (relative humidity <5%). Illuminated current as a function of forward bias characteristics of the Al_0.2_Ga_0.8_As ^55^Fe radioisotope X-ray-photovoltaic microbattery were investigated over the temperature range −20 °C to 50 °C. Forward bias measurements from 0 V to 0.5 V were made in 0.005 V increments using a Keithley 6487 picoammeter/voltage source. The uncertainty associated with a single current measurement reading was 0.3% of their values plus 400 fA, while the uncertainty associated with the applied biases was 0.1% of their values plus 1 mV^28^. It has to be noted that the uncertainty in current readings decreased for a set of measurements taken at the same Keithley working conditions (e.g. no variations in electrical connections, temperature); in this situation fittings on the experimental data give more appropriate current uncertainty values. Exponential fittings on the measured Al_0.2_Ga_0.8_As currents as a function of forward bias characteristics have been performed, current uncertainty <0.05 pA was estimated. Figure 1a shows the measured illuminated characteristics. Comparable results were obtained for both devices but for clarity only the results from one device, D1, are shown in the Fig. 1a. As shown in Fig. 1a, the softness in the knee of the measured current as a function of applied forward bias decreased when the temperature increased. Figure 1b shows dark and illuminated current characteristics as a function of forward bias for D2 at 20 °C.

**Figure 2:**
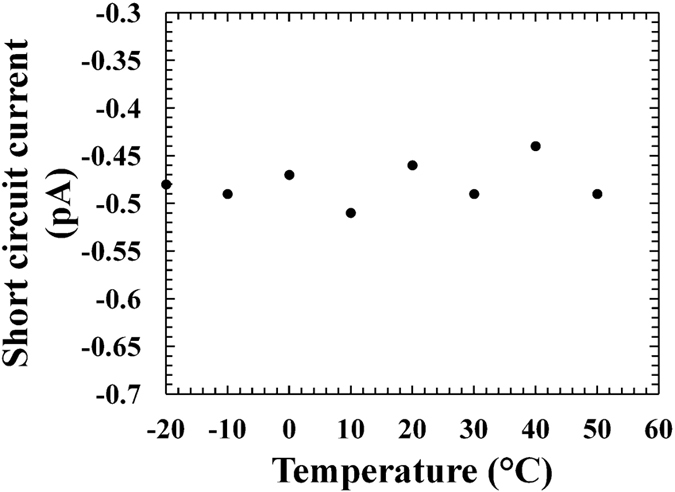
Short circuit current as a function of temperature for Al_0.2_Ga_0.8_As ^55^Fe radioisotope microbattery.

The short circuit current (*I*_*SC*_) is defined as the current through the device when the voltage across it is zero. At every temperature, the experimental short circuit current values were obtained as the interception point of the curves in Fig. 1a on the vertical axis. In Fig. 2 the *I*_*SC*_ values measured for D1 are reported as a function of temperature; comparable results were found for D2.

For a simple p-n diode, the short circuit current is proportional to the rate of optical (X-ray) carrier generation and the carrier diffusion length^29^. When the temperature is increased, it is expected that the optically (X-ray) generated carrier rate should increase because of the lower electron-hole pair creation energy at higher temperatures, whilst the carrier diffusion lengths should decrease because of the increased scattering with phonons. Although measurements of the electron-hole pair creation energy are yet to be reported for Al_0.2_Ga_0.8_As, it was reasonable to assume a decrease of electron-hole pair creation energy at high temperature (Barnett *et al*. reported a reduction in the average energy consumed in the generation of an electron-hole pair at high temperatures for Al_0.8_Ga_0.2_As illuminated with X-rays^30^). In Fig. 2, an almost flat trend was observed for the short circuit current with temperature; this was possibly due to the increased optical (X-ray) carrier generation being compensated by the decrease in the carrier diffusion lengths when the temperature was increased. The values of short circuit current observed for the Al_0.2_Ga_0.8_As ^55^Fe radioisotope microbattery were much lower than that previously reported for GaAs (~7 pA at −20 °C)^13^ and Al_0.52_In_0.48_P (~1.15 pA at −20 °C)^14^ ^55^Fe radioisotope microbatteries. This smaller short circuit current in the presently reported Al_0.2_Ga_0.8_As microbattery was mainly attributed to the poorer crystalline quality of the Al_0.2_Ga_0.8_As structure with respect to the GaAs and Al_0.52_In_0.48_P detectors. In comparison with the GaAs microbattery, the Al_0.2_Ga_0.8_As converter layer also had a thinner i-layer (3 μm instead of 10 μm). Further analysis need to be conducted such to confirm the crystalline quality problem in the fabricated Al_0.2_Ga_0.8_As device.

**Figure 3:**
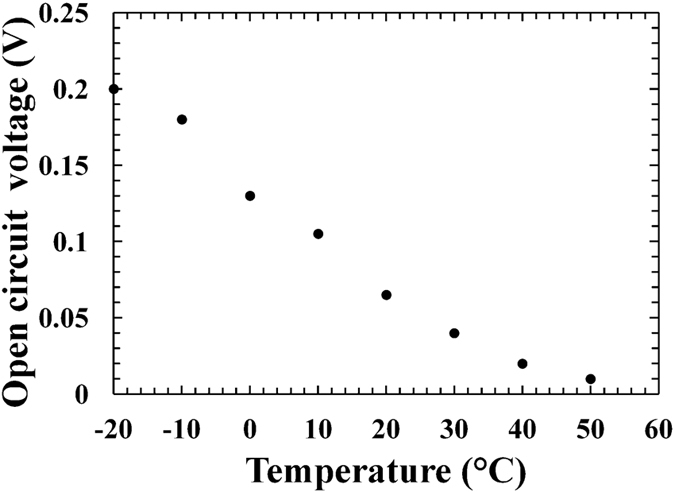
Open circuit voltage as a function of temperature for Al_0.2_Ga_0.8_As ^55^Fe radioisotope microbattery.

The open circuit voltage (*V*_*OC*_) is defined as the voltage across the device when the current is zero. At each temperature, the open circuit voltage was obtained as the interception point of the curves in Fig. 1a on the horizontal axis. In Fig. 3, the *V*_*OC*_ measured for D1 are reported as a function of temperature; similar results were found for D2.

For a simple p-n diode, the open circuit voltage is given by Equation 1,


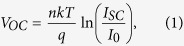


where *I*_*SC*_ is the short circuit current, *I*_*0*_is the saturation current, *T* is the temperature, k the Boltzmann’s constant, q the electronic charge and *n* the ideality factor^11^. Since *I*_*0*_ increases exponentially with temperature due to the thermal excitation of carriers in the conduction band; *V*_*OC*_ is expected to decrease linearly when the temperature increases^11^. As expected and shown in Fig. 3, the open circuit voltage (*V*_*OC*_) decreased with temperature in the studied Al_0.2_Ga_0.8_As devices. The observed values of open circuit voltage of the presently reported microbattery were lower than those of the previously reported GaAs^13^ and Al_0.52_In_0.48_P^14^ ^55^Fe radioisotope microbatteries. At −20 °C, for example, the Al_0.2_Ga_0.8_As ^55^Fe radioisotope microbattery had a *V*_*OC*_ of 0.2 V; whilst the GaAs and Al_0.52_In_0.48_P microbatteries had *V*_*OC*_ of 0.3 V and 1 V, respectively. This result can be explained by the lower *I*_*SC*_ and possibly higher *I*_*0*_ in the Al_0.2_Ga_0.8_As structures. Material issues, such as the presence of traps in the structure, could be the one cause of the poor Al_0.2_Ga_0.8_As microbattery performance: trapped carriers from previous events can enhance the devices dark current resulting in higher *I*_*0*_ observed. The increased dark current also affected the number of photo-generated carriers detected by the devices: the photo-generated carriers can recombine with the charges trapped in the device resulting in lower *I*_*SC*_^31^.

**Figure 4:**
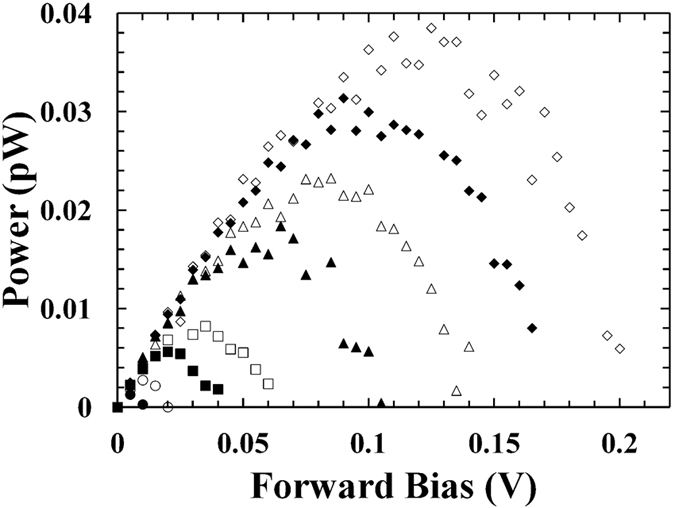
Output power as a function of applied forward bias for D1 at different temperatures. The temperatures studied were 50 °C (filled circles), 40 °C (empty circles), 30 °C (filled squares), 20 °C (empty squares), 10 °C (filled triangles), 0 °C (empty triangles), −10 °C (filled rhombuses) and −20 °C (empty rhombuses).

**Figure 5:**
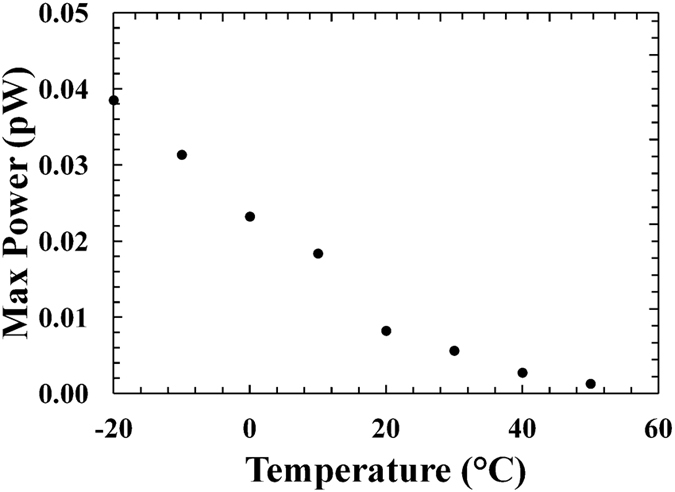
Experimental maximum output power as a function of temperature for D1.

At every investigated temperature, the output power (*P*) from each Al_0.2_Ga_0.8_As photodiode was computed as *P* = *IV*. When the bias was increased, the output power increased to a maximum (*P*_*m*_) and then decreased. Figure 4 shows the output power from D1, similar results were obtained for D2. Figure 5 shows the magnitude of the maximum output power achieved for D1 at different temperatures. Similar results were found for D2.

As the temperature was increased, the magnitude of the maximum output power decreased. This behaviour was expected considering that the dependence of the maximum output power (*P*_*m*_) on the open circuit voltage^32^. In a photovoltaic cell, the maximum output power is given by Equation 2:


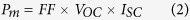


where *V*_*OC*_ is the open circuit voltage, *I*_*SC*_ is the short circuit current, and *FF* is a parameter called the fill factor. The fill factor is the measure of the squareness of the current as a function of voltage characteristic presented in Fig. 1a.

A maximum output power of 0.04 pW, corresponding to 0.017 μW/Ci (ratio between the maximum output power, 0.04 pW, and the number of photon expected on the detector, 0.08 × 10^6^ s^−1^ = 2.26 × 10^−6 ^Ci), was observed from the Al_0.2_Ga_0.8_As cell D1 at −20 °C.

The number of photons per second emitted in any direction by the source was estimated knowing the activity of the source and the emission probabilities of Mn Kα and Mn Kβ X-rays from ^55^Fe (0.245 and 0.0338, respectively^25^); it was found that 6.4 × 10^7^ photons per second are emitted by the ^55^Fe radioactive source. Of these 6.4 × 10^7^ photons per second, only half are emitted in the direction of the devices (we assumed that half of the photons were lost because they were emitted up). The number of photons per second on the devices (0.08 × 10^6^ s^−1^) was estimated knowing the number of photons per second emitted by the source towards the devices (3.2 × 10^7^ s^−1^), the thickness of the radioisotope X-ray source’s Be window (0.25 mm) and the geometry of the source and detectors. The ratio between the area of the devices (0.13 mm^2^) and the area of the radioactive ^55^Fe source (28.27 mm^2^) was calculated to be 0.0046. The number of photons on the detector was estimated by multiplying 0.0046 for the number of photons per seconds transmitted trough the X-ray source’s Be window (1.8 × 10^7^ s^−1^).

The observed maximum output power value is much lower than the maximum output powers previously observed using GaAs cell (~1 pW)^13^ and Al_0.52_In_0.48_P cell (~0.62 pW)^14^ ^55^Fe radioisotope microbatteries, at the same temperature. This was due to the smaller open circuit voltage and short circuit current values measured in the Al_0.2_Ga_0.8_As ^55^Fe radioisotope microbattery. The Al_0.2_Ga_0.8_As ^55^Fe radioisotope microbattery was of a two cell design (i.e. two photodiodes) such that the output power of the cells could be combined, giving a total microbattery output power of 0.07 pW at −20 °C. This was still less than the single cell GaAs and single cell Al_0.52_In_0.48_P ^55^Fe microbatteries previously reported.

The internal conversion efficiencies (*η*) of the Al_0.2_Ga_0.8_As photodiodes were investigated in the temperature range studied. Dividing the measured single cell maximum output power (*P*_*m*_) by the maximum power (*P*_*th*_) obtainable from the X-ray photons usefully absorbed by the device, the internal conversion efficiency of each Al_0.2_Ga_0.8_As device was calculated (equation 3).


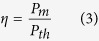


*P*_*th*_ was calculated using equation 4


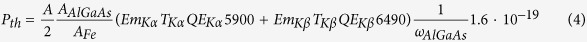


where *A* is the activity of the ^55^Fe radioactive source (230 MBq), *A*_*Fe*_ area of the ^55^Fe radioactive source (28.27 mm^2^), *A*_AlGaAs_ area of the Al_0.2_Ga_0.8_As detector (0.13 mm^2^), *Em*_*Kα*_and *Em*_*Kβ*_ the emission probabilities of Mn Kα and Mn Kβ X-rays from ^55^Fe (0.245 and 0.0338, respectively^33^), *T*_*Kα*_and *T*_*Kβ*_ the transmission probabilities of Mn Kα and Mn Kβ X-rays through the 0.25 mm Be window (0.576 and 0.667, respectively^25^,^27^), *QE*_*Kα*_ and *QE*_*Kβ*_ the quantum efficiency values calculated in section “Material and Method A”, *ω*_*AlGaAs*_ the Al_0.2_Ga_0.8_As electron-hole pair creation energy (4.4 eV, 2.5 times the bandgap). In equation 4 the activity of the ^55^Fe radioactive source was halved because we assumed that half of the X-ray photons were lost since they were emitted up. *P*_*th*_ was found to be 4 pW.

**Figure 6:**
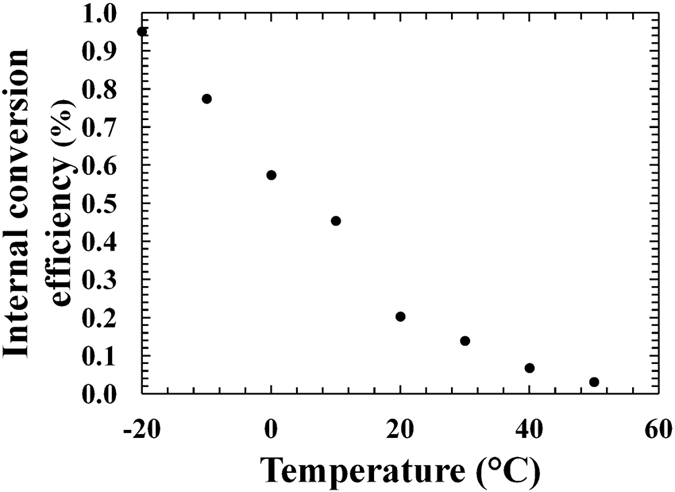
Internal conversion efficiency (*η*) as a function of temperature for D1.

In Fig. 6, the internal conversion efficiency as a function of temperature for D1 is shown. Decreasing the temperature, the efficiency increased, in accordance with the maximum output power results presented in Fig. 5. At −20 °C, internal conversion efficiencies as high as 0.95% and 0.85% were observed for D1 and D2, respectively.

Although the bandgap of Al_0.2_Ga_0.8_As (1.67 eV at room temperature^15^) was greater than that of GaAs (1.42 eV at room temperature^34^), the internal conversion efficiency in the Al_0.2_Ga_0.8_As ^55^Fe radioisotope microbattery was lower than that observed in the GaAs ^55^Fe radioisotope microbattery^13^ at the same temperature. Material quality issues such as the presence of traps in the fabricated structure could explain the poorer performance observed. The presence of traps in AlGaAs devices have been previously reported by other researchers^35^,^36^,^37^,^38^. Traps can cause polarisation effects that may contribute to the device function^39^: the charge generated by the X-ray radiation can be trapped, building up space charges in the detector, which collapses the electric field and results in device degradation. The resulting change in the electric field has consequences for the electron transport within the semiconductor: the electron transit time in the detector depletion region increases, whilst the lifetime decreases due to recombination process. This causes a lower charge collection efficiency that reduced the photocurrent observed. Further analysis needs to be conducted such to confirm the crystalline quality problem of the fabricated Al_0.2_Ga_0.8_As device.

In the next generation of Al_0.2_Ga_0.8_As ^55^Fe radioisotope microbatteries, the maximum output power and consequently the internal conversion efficiency of the microbattery prototype (in accordance with what is expected by its wide bandgap) will have to be increased if Al_0.2_Ga_0.8_As is to become a realistic prospect for nuclear microbattery applications. The quality of the material must be improved to maximise the performance of the converter device. The system design must also be improved to increase the number of photons impinging upon the Al_0.2_Ga_0.8_As devices. The fraction of photons emitted by the ^55^Fe radioisotope X-ray source that impinged upon the devices was estimated knowing the activity of the source, the emission probabilities of the ^55^Fe Mn Kα and Mn Kβ X-rays (0.245 and 0.0338, respectively^33^), the thickness of the radioisotope X-ray source’s Be window (0.25 mm), and the geometry of the source and detectors. It was computed that only 0.26% of the photons emitted by the ^55^Fe radioisotope X-ray source were incident on the conversion devices.

## Conclusion

In this paper, a prototype Al_0.2_Ga_0.8_As ^55^Fe radioisotope microbattery has been reported for the first time: a 230 MBq ^55^Fe radioisotope X-ray source was coupled to p^+^-i-n^+^ Al_0.2_Ga_0.8_As mesa photodiodes to achieve the direct conversion of nuclear energy into electrical energy. The performances of the fabricated prototype are described; in particular, the changes in key microbattery parameters are reported as a function of temperature. As the temperature was increased, the open circuit voltage, maximum power and internal conversion efficiency decreased. An open circuit voltage of 0.2 V was observed in a single cell Al_0.2_Ga_0.8_As p^+^-i-n^+^ mesa structure at −20 °C. An internal conversion efficiency of 0.95% was observed at −20 °C, taking into account attenuation from contacts and dead layer. Combining the output powers extracted from both the Al_0.2_Ga_0.8_As photodiodes, a total microbattery maximum output power of 0.07 pW was measured at −20 °C. Further studies need to be conducted such to assess the suitability of the Al_0.2_Ga_0.8_As material performance as X-ray energy conversion material, this will allow to conclude which of the above microbattery parameters are limited by the material itself and which depend on fabrication procedure and structure design.

## Additional Information

**How to cite this article**: Butera, S. *et al*. AlGaAs ^55^Fe X-ray radioisotope microbattery. *Sci. Rep.* **6**, 38409; doi: 10.1038/srep38409 (2016).

**Publisher's note:** Springer Nature remains neutral with regard to jurisdictional claims in published maps and institutional affiliations.

## Figures and Tables

**Table 1:** Layer details of the Al_0.2_Ga_0.8_As X-ray-photodiode.

Layer	Material	Thickness (μm)	Dopant	Dopant Type	Doping density (cm^−3^)
1	Ti	0.02			
2	Au	0.2			
3	GaAs	0.01	Be	p^+^	1 × 10^19^
4	Al_0.2_Ga_0.8_As	0.5	Be	p^+^	2 × 10^18^
5	Al_0.2_Ga_0.8_As	3	undoped		<10^15^
6	Al_0.2_Ga_0.8_As	1	Si	n^+^	2 × 10^18^
7	Substrate n^+^ GaAs				
8	Au	0.2			
9	InGe	0.02			
